# Is DNA repair controlled by a biological logic circuit?

**DOI:** 10.1007/s12064-021-00360-8

**Published:** 2022-01-01

**Authors:** Philip G. Penketh

**Affiliations:** Department of Pharmacology, Yale University School of Medicine, New Haven, CT 06520 USA

**Keywords:** Logic control, Digital control, Biological clock, Oscillator, Phosphorylation, Methylation, DNA repair

## Abstract

The possible utilization of biological logic circuit(s) in the integration and regulation of DNA repair is discussed. The author believes this mode of regulation likely applies to many other areas of cell biology; however, there are currently more experimental data to support its involvement in the control of DNA repair. Sequential logic processes always require a clock to orchestrate the orderly processing of events. In the proposed hypothesis, the pulses in the expression of p53 serve this function. Given the many advantages of logic type control, one would expect that in the course of ~ 3 billion years of evolution, where single cell life forms were likely the only forms of life, a biological logic type control system would have evolved to control at least some biological processes. Several other required components in addition to the ‘clock’ have been identified, such as; a method to temporarily inactivate repair processes when they are not required (e.g. the reversible inactivation of MGMT, a suicide repair protein, by phosphorylation); this prevents complex DNA repair systems with potentially overlapping repair functions from interfering with each other.

## Introduction

I suspect that many cellular processes are controlled by biological logic type control mechanisms. However, in this article, I am going to focus entirely on DNA repair, for which, I believe, there is mounting evidence for this type of control in the cell. This hypothesis requires the presence of a 'clock' and thus provides an explanation for why the levels of the p53 protein (commonly known as the guardian of the genome) pulses with a beat frequency of approximately 2.5 cycles per hour (Porter et al. 2016), why the different DNA repair machineries do not interfere with each other's actions in normal cells, why MGMT (O^6^-methylguanine-DNA methyltransferase, an enzyme that can only repair a single DNA lesion per MGMT molecule (Gerson 2002)) should be reversibly inhibited by phosphorylation/dephosphorylation (Mullapudi et al. 2000), and why there are more than 4,000 known protein kinases and 15,000 sites on cellular proteins in mammalian cells that are known to undergo phosphorylation/dephosphorylation (Yang et al. 2008).

In the late 1990s, I was working as part of Professor Alan C. Sartorelli's research group, developing tumour tissue targeted DNA damaging anticancer drugs (Shyam et al. 2015; Penketh et al. 2020). This necessitated having some knowledge of DNA repair mechanisms and cancer cell biology. Around this same time, I had developed an interest in electronic circuit design and had produced a number of simple logic control circuits for various laboratory and hobby automotive purposes. It was probably this combination of circumstances that made me think, at that time, about the possible role of biological analogues to electronic logic/digital decision making circuits in the control of cells. Twenty two years later, I have finally found the time to put pen to paper and detail some of these early ideas. In the intervening years, I have seen more evidence in the literature arise that supports these contentions. Given the many advantages of digital control over analogue control systems, one would expect that in the course of the approximate 3 × 10^9^ years of evolution, where single cell life forms were likely the only forms of life on Earth, a biological logic/digital type control system should have evolved to control at least some biological processes. Biology does not have the ability to go back to the drawing board like engineers do, and so these systems would have to be built upon and developed from earlier control systems, or arisen directly. I thought about this possibility from two main points of view: (i) how would one build digital/logic circuits using already well understood biological control mechanisms such as protein phosphorylation, (ii) which areas of cell biology would most likely benefit from the adoption of digital/logic control circuits. This hypothesis required a clock to produce a series of regular pulses as a cornerstone component. At the time that I proposed this model to my colleagues, and detailed this hypothesis in my laboratory notebook, this clock (the pulses of the p53 clock) was still a decade from its discovery (Zhang et al. 2009).

In the 1980s and 1990s familiar everyday analogue systems were being replaced with digital systems. Analogue audio in the form of vinyl records and audio cassette tapes were replaced with CDs, and VHS video tapes were replaced by DVDs. However, the switch from analogue fuel delivery systems such as carburetors to much more sophisticated computer (digital/logic circuit) control in the automotive industry is probably the most apt analogue to living organisms. Cells 'burn' hydrogen and carbon containing fuels to provide them with energy just as cars burn hydrogen and carbon containing fuels for propulsion.

Using more sophisticated computer (digital/logic circuits) control allows for much more precise fuel control, and options simply unavailable to analogue carburetors, such as making adjustments for fuel quality/octane rating, different atmospheric conditions (e.g. barometric pressure, humidity, etc.), cutting fuel to cylinders when descending hills for greater fuel economy (depending on the input values from various sensors), or going into a super green non-polluting mode when the car is being monitored at an emissions station, then reverting to a polluting performance orientated machine, when no longer being watched, as employed by Volkswagen engineers resulting in their 2017 emissions scandal, when finally discovered (https:, , en.wikipedia.org, wiki, Volkswagen_emissions_scandal. xxxx). In fact, it would be very surprising that cells after eons of competitive selection pressure would have failed completely to take advantage of this more sophisticated type of regulation.

## Discussion

### How could one build digital/logic circuits using known biological processes such as protein phosphorylation

**Fig. 1:**
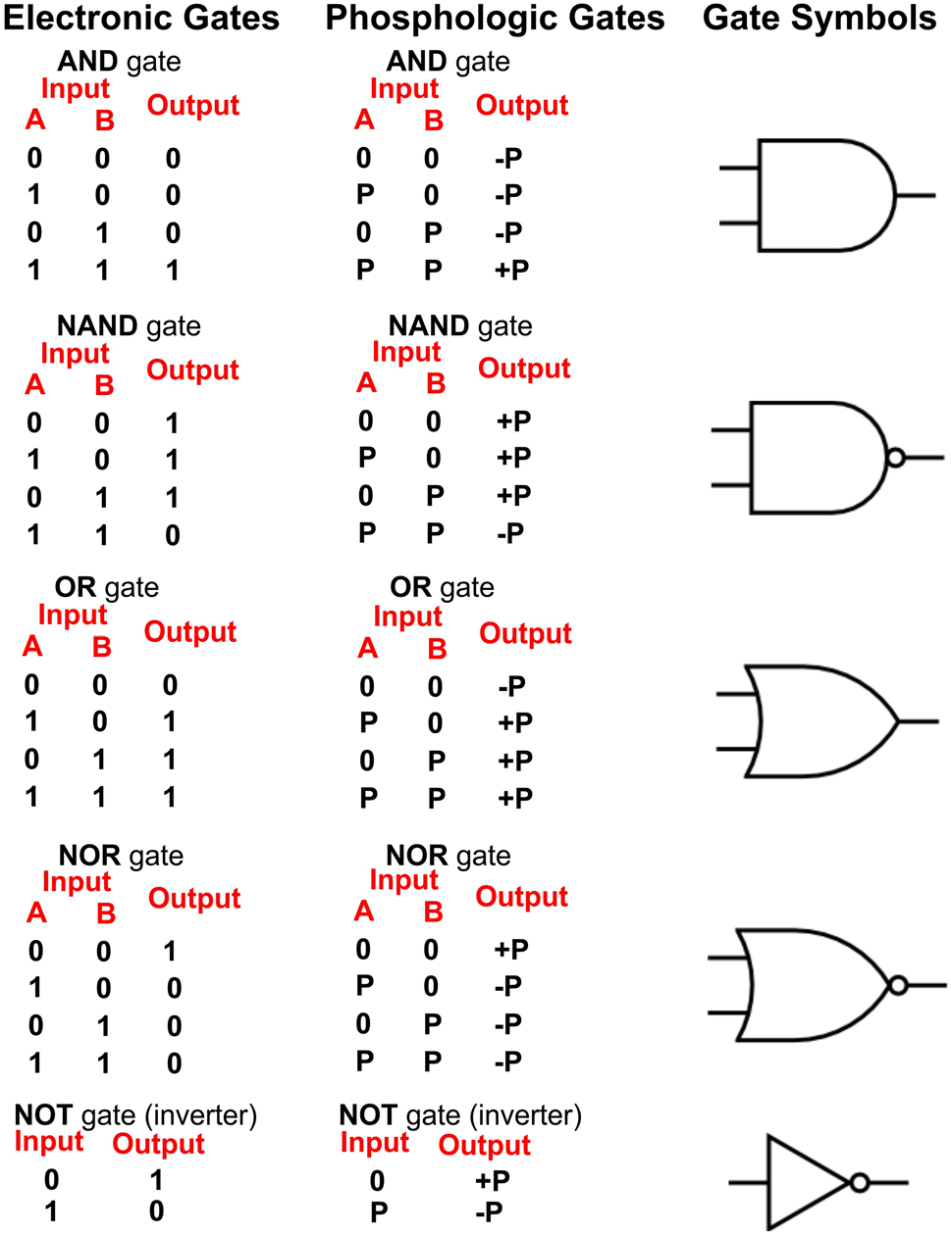
Table giving important electronic, and their analogous phosphologic gates, together with their symbols. Note: all the electronic gates have two extra connections, not involved in information processing, these supply the energy for the gates operation. However, these connections are not shown in Fig. 1 for reasons of clarity. Similarly in the analogous phosphologic gates the source of the energy in the form of ATP production is also not shown

If we assumed cellular decision making was not an analogue process but occurred in a manner analogous to electronic logic circuits, we would have to view protein phosphorylation (a well-documented enzyme activity regulatory process) as resulting in an all or nothing event with all copies of a particular protein rapidly becoming phosphorylated or dephosphorylated at a particular amino acid site, as opposed to a progressive increase in activity (or decrease in activity) as the cellular population of this protein molecule became progressively modified by phosphorylation/dephosphorylation. (Note: this would not be observed as an all or nothing event in a population of cells unless they were all metabolically synchronized in some way. Furthermore, several co-existing logic circuits could exist in the various membrane bound intracellular compartments that were not necessarily in phase). These cellular logic circuits could allow individual cells to each behave very much like integrated logic circuits or small CPUs. Thus, individual cells could perform complex metabolic data processing/decision making and counting functions. First let us see how we could model the electronic gates commonly used in the construction of CPUs using protein kinases (a protein kinase is an enzyme that adds a phosphate group usually from ATP to a specific amino acid residue in its substrate protein), and protein phosphatases (a protein phosphatase is an enzyme that removes a phosphate group from a specific phosphorylated amino acid residue in its substrate protein). Then see how their behaviour would compare to their electronic counterparts. Figure 1 gives the symbols and truth tables for some of the common electronic logic gates that are used to produce CPUs. A '**1**' indicates a high electrical potential (usually about + 3 V) and a '**0**' indicates an absence of electrical potential (usually a value close to 0 V).

These electronic gates can be arranged to perform various complex functions. We could generate functionally equivalent gates using protein kinases, and protein phosphorylases. I will use the same gate symbols but the input and outputs are different although they can still be represented by '**1**'s and '**0**'s. If we considered a protein phosphorylation/dephosphorylation based **AND** gate, (I have used the abbreviation + **P** to indicate phosphorylation, and **-P** to indicate dephosphorylation.) + **P** only at an amino acid corresponding to site/input **A** would result in no change in its output (**+ P** activity towards one of its substrate proteins). Similarly, + **P** solely at an amino acid corresponding to site/input **B** would result in no change in its output (**+ P** activity towards one of its substrate proteins), and **-P** at both **A** and **B** would also result in no output activity. However, if this protein is + **P** at both sites **A** and **B** this protein now expresses its activity, that is the + **P** of its substrate proteins. This would result in a protein/metabolic **AND** gate (**AND** gates with more than 2 inputs are of course possible). This **AND** gate would be connected to other gates by the substrate specificity of the **AND** gate enzyme, and because it would be in a homogenous solution there would be no problem having this gate connected to a vast number of other biological gates and effectors. All the known electronic gates could be similarly modelled, and directly analogous biological circuits to electronic logic circuits (memory, counting, decision making, etc.) could be constructed using combinations of these biological gates (Fig. 1). A variant of the **NOR** gate is the **exclusive NOR** gate. In our phosphologic circuits, the **exclusive NOR** gate would differ from a **NOR** gate in that when both **A** and **B** were phosphorylated, the **exclusive NOR** gate would exhibit + **P** activity, instead of **-P** activity. One other useful component we should introduce is an inverter (also known as a **NOT** gate) this only has one input site; when it is + **P** it becomes a **-P** to its substrate proteins, when it is **-P** it becomes a + **P** to its substrate proteins. It is a direct analogue of the electronic logic gate with the same name (in Fig. 1 I have used the equivalent symbols for both electronic and biological protein gates). In the electronic **NOT** gate, a high voltage input gives a low voltage output and vice versa. The truth tables, symbols, and outputs/actions are illustrated in Fig. 1 for both the biological and electronic logic gates.

Note: all the electronic gates have two extra connections, not involved in information processing, these supply the energy for the gates operation. However, these connections are not shown in Fig. 1 for reasons of clarity. Similarly, in the analogous phosphologic gates, the source of the energy in the form of ATP production is also not shown. The phosphorylation circuits described (unlike their electronic counterparts) would essentially involve latching switches and so they would require a **-P** activity to remove the phosphate groups to reset the gate. This **-P** activity could either be intrinsic to the protein itself, or a specific or generic additional **-P** activity, that would be required for non-permanent circuit commitment. The intrinsic latching nature of these gates could make them very energy efficient. If a cell or cellular compartment containing the circuit were to divide, both daughter cells would inherit the same circuit in the same functional logic state (unless one of the component proteins was present in a very low copy number like 1).

Logic circuits have certain unique problems to which analogue circuits are generally immune; if a disallowed state occurs, it can result in freezing/lock-up. It is possible that certain mutations could result in biological logic circuits that are more prone to such freezing/lock up, or perfectly normal cells could enter a disallowed state due to abnormal inputs and become locked, possibly in a proliferative state and result in certain cancerous phenotypes. If these cells were 'rebooted', one might expect them to then behave relatively normally. With biological logic circuits, it would be possible to have several independent circuits in the same sub-cellular compartment using different types of covalent protein modifications. Although + **P** is the most well known, many other types occur. Protein methylation/demethylation is somewhat analogous to protein + **P/-P**. When a protein is + **P,** a charged phosphate group is added to the molecule which can then form strong ionic bonds and hydrogen bonds causing a large conformational change (and the opposite would occur on **-P**). In the case of protein methylation at a protein carboxyl group, a charged group (which may be involved in strong ionic bonds and hydrogen bonds) becomes a neutral group and its ability to form ionic bonds is lost resulting in a protein conformational change. These independent circuit pathways could of course intersect. A review of enzyme activity regulation by methylation and demethylation events is described in Bedford (2006).

To ascertain the possibility of such phenomena, we need to find the basic metabolic logic circuit components. Electronic CPUs have a clock that generates a square wave. In CPUs, the clock rate typically refers to the square wave frequency, which is used to synchronize the processing of data. In a biological data processor, a clock would still be required. Therefore, we would need to find evidence for at least the following components:

1. Biochemical clock of appropriate frequency (p53). Many DNA repair processes are highly complex involving multiple steps and proteins. If multiple repair attempts by a system are also allowed (see later), this would result in the overall process taking several hours, and this would require a clock cycle time to be of the order of one cycle per several hours.
2. Biochemical **AND**, **NAND**, **OR**, and **NOR** gates etc.
3. Input signals need to be digital or converted to a digital form (note: the insulin receptor is known to be like this; it is a latching switch that locks on and needs to be reset (all or nothing, a '1' or a '0'), the activated insulin receptor/switch initiates a cascade of different protein activation or deactivation as a result of their + **P/-P** (Boucher et al. 2014).
4. If a biological logic system existed based on protein + **P/-P** a large number of proteins with + **P** activity and **-P** activity, and target proteins to undergo + **P/-P** would have to exist. There is very strong evidence for this contention as greater than 4000 protein kinases are encoded by the human genome, and more than 15,000 human cellular proteins are thought to undergo + **P/-P** (Yang et al. 2008).

I have detailed above how an electronic, and how a + **P/-P AND** gate would function and differ. Let us consider biological + **P/-P** analogues of some simple circuits utilizing these gates. I have coined the term 'phosphologic' to describe this class of potential biological logic gates, and I will use the same symbols for the phosphologic gates as their equivalent electronic logic gates. I will also restrict this paper to phosphologic gates, even though, as mentioned earlier, other covalent modifications of proteins (carboxymethylation), and non-covalent, purely conformational gates are of course possible. These phosphologic gates need not be each composed of a single polypeptide chain but could be a polymeric protein molecule. More complex gates employing more than two inputs, or that function as buffers or inverters are of course possible, but we will stick with the gates described above. The truth tables for the phosphologic gates would of course be identical to their digital counterparts. In this paper, I will focus on the potential role of phosphologic gates and circuits in the orchestration of DNA repair/maintenance. DNA itself is essentially a digital base 4 code for the production and regulation of protein production. It would be surprising if life at this point would switch to an analogue system to repair and maintain the digital genetic code. Let us examine some simple potential phosphologic circuits, then study the biological observations of others to find evidence for the existence of such phosphologic circuits in their observations. It should be noted that cancer cells usually possess a defect in their DNA repair (Loeb and Loeb 2000), leading to the acquisition of the subsequent mutations required to produce a cancerous phenotype. Thus, the study of DNA repair in cancer cells likely will not accurately reflect the true overall picture of DNA repair in normal healthy cells.

### Simple phosphologic circuits (direct analogues of digital logic circuits)

**Fig. 2:**
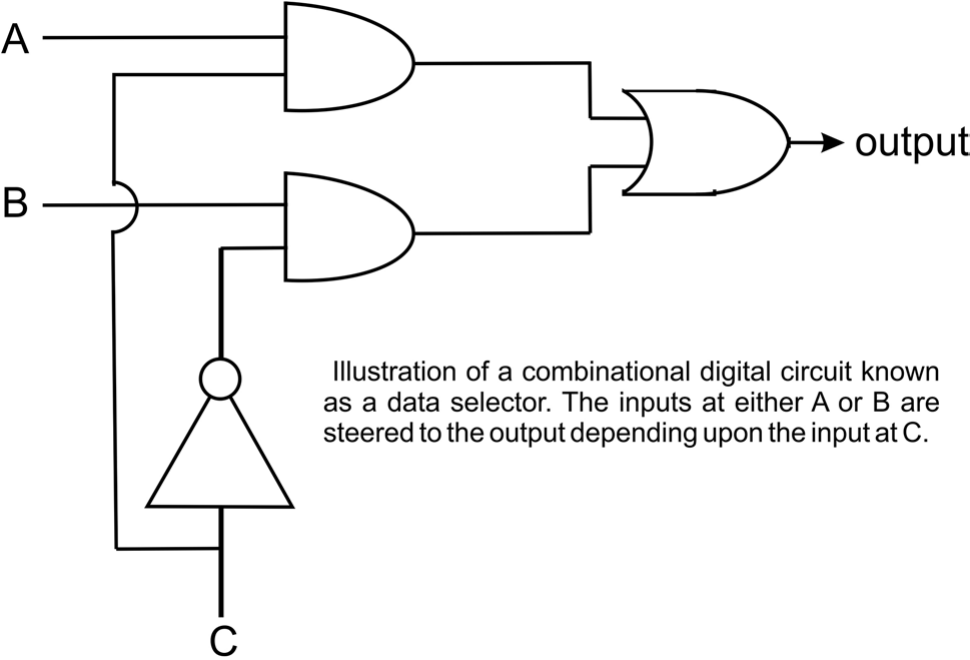
Illustration of a combinational digital circuit known as a data selector. The inputs at either A or B are steered to the output depending upon the input at C

There are two basic classes of decision making circuits, combinational and sequential (sequential circuits require a clock, *i.e.* p53). An example of a phosphologic combinational circuit is a data selector. This is illustrated in Fig. 2. If 'C' the input to the inverter is dephosphorylated, the signal at 'A' (either phosphorylated or dephosphorylated) goes to the output. If 'C' the input to the inverter is phosphorylated, the signal at 'B' (either phosphorylated or dephosphorylated) goes to the output. The behaviour of this circuit would be identical whether it was an electronic data selector circuit or a phosphologic data selector circuit. In the phosphologic circuit, the connections are not wires they are just the substrate specificities of the + **P/-P** proteins. In the phosphologic circuit, the inputs at 'A' and 'B' could be the + **P** outputs of two different receptors. These inputs either at 'A' or 'B' could be steered to the output (connected to a further decision making circuit, or to gene expression) depending upon the input at 'C'. The control at 'C' could depend on the status of the cell, or environmental factors like the availability of different carbon sources, or bases to synthesize DNA, or the output of another phosphologic circuit, etc. Therefore, under one set of conditions, the output depends solely upon receptor 'A', and under a second set of conditions, the output depends solely upon receptor 'B'. Details on design and operation of the various electronic logic gates and their use in logic circuits can be found in various computer science course textbooks such as (Marcovitz 2005).

**Fig. 3:**
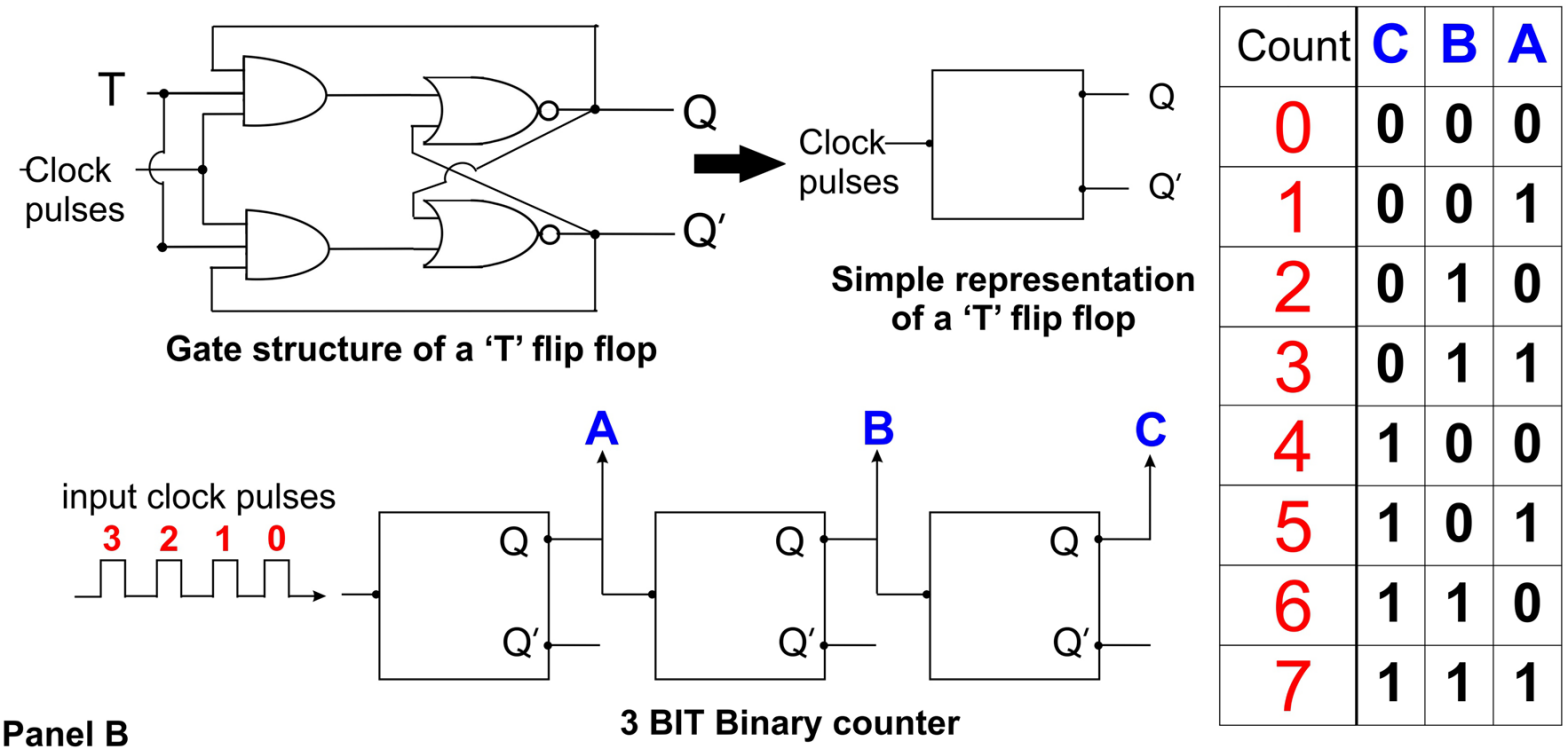
Top left: gate arrangement to produce a ‘T’ flip flop. Top center: Box representation of a ‘T’ flip flop. Lower center figure connection of 3 ‘T’ flip flops to construct a 3 BIT binary counter. Right hand table: Binary output count (at A, B, and C) of the pulses fed into the 3 BIT counter. One additional pulse after pulse 7 would reset the counter to 0. In a phosphologic circuit the input pulses would be from p53, and the output binary count could control the number of repair attempts by a particular repair system, or schedule the sequence in which the repair systems operate

Electronic digital counting and sequential circuits (all built from the gates described above), where BITs (Binary Digit) of information are processed sequentially, all require a clock which progressively advances the information through the circuit with each clock cycle (Fig. 3). When the p53 pulse was published in 2009 (7) and the necessary clock was discovered, this finding convinced me that this hypothesis had a firm foundation supported by experimental evidence. Errors with the clock would play havoc with data processing (DNA repair) and would explain why p53 is found to be mutated in many cancers.

Using the above described phosphologic gates, we could build phosphologic circuits that could count events, allow a predetermined number of DNA repairs attempts with a particular repair system, before switching to another DNA repair process/set of repair proteins, and define the sequence of repair events, and store information (like flash memory) such as which repair systems have already been tried (Fig. 3). In the cell, the repair machinery would function sequentially with (to borrow a few terms from baseball) the currently active repair process being 'Up', the next in line being 'On deck' and the next being 'In the hole' and thus would require a clock with the appropriate time base frequency. This would also explain why MGMT, a one shot repair protein, should be inactivated by phosphorylation as this action places it 'On deck' or 'In the hole'. For a review of the details of the various classes of DNA machinery see (Turgeon et al. 2018).

**Fig. 4:**
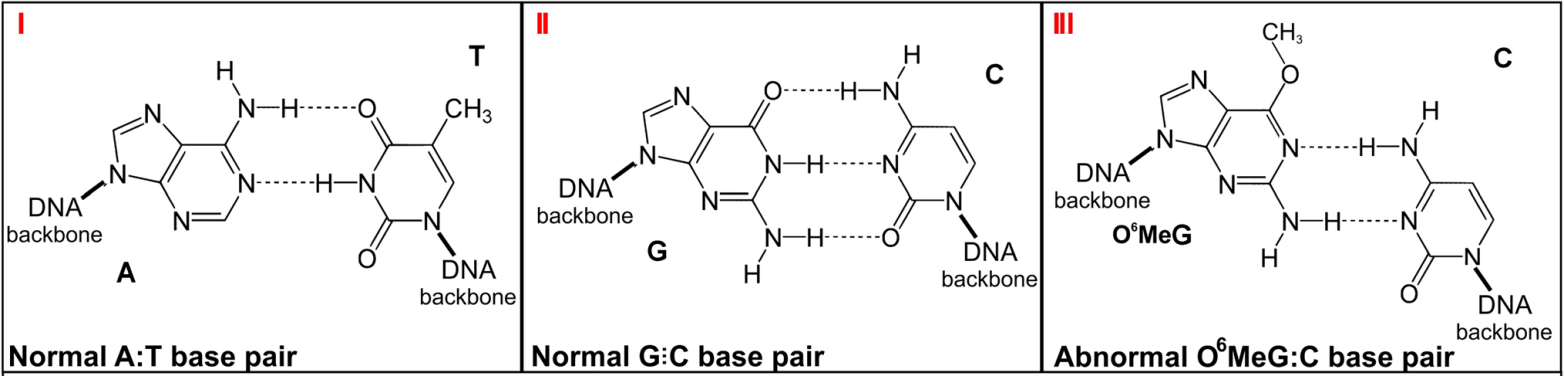
Normal, and abnormal O6MeGC base pairs in DNA and MMR futile cycles. Panel I normal AT base pair; Panel II normal GC base pair; Panel III abnormal O6MeGC base pair, showing some characteristics of both the AT, and the GC base pairs. Mismatch repair correctly recognizes the O6MeGC base pair as an erroneous base pair, but sees the C as the error, it then cuts out the C, only to replace it with another C, thus entering into a futile cycle. MGMT reacts with O6MeG removing the methyl group, restoring the G, and thus escaping the futile cycle

**Fig. 5:**
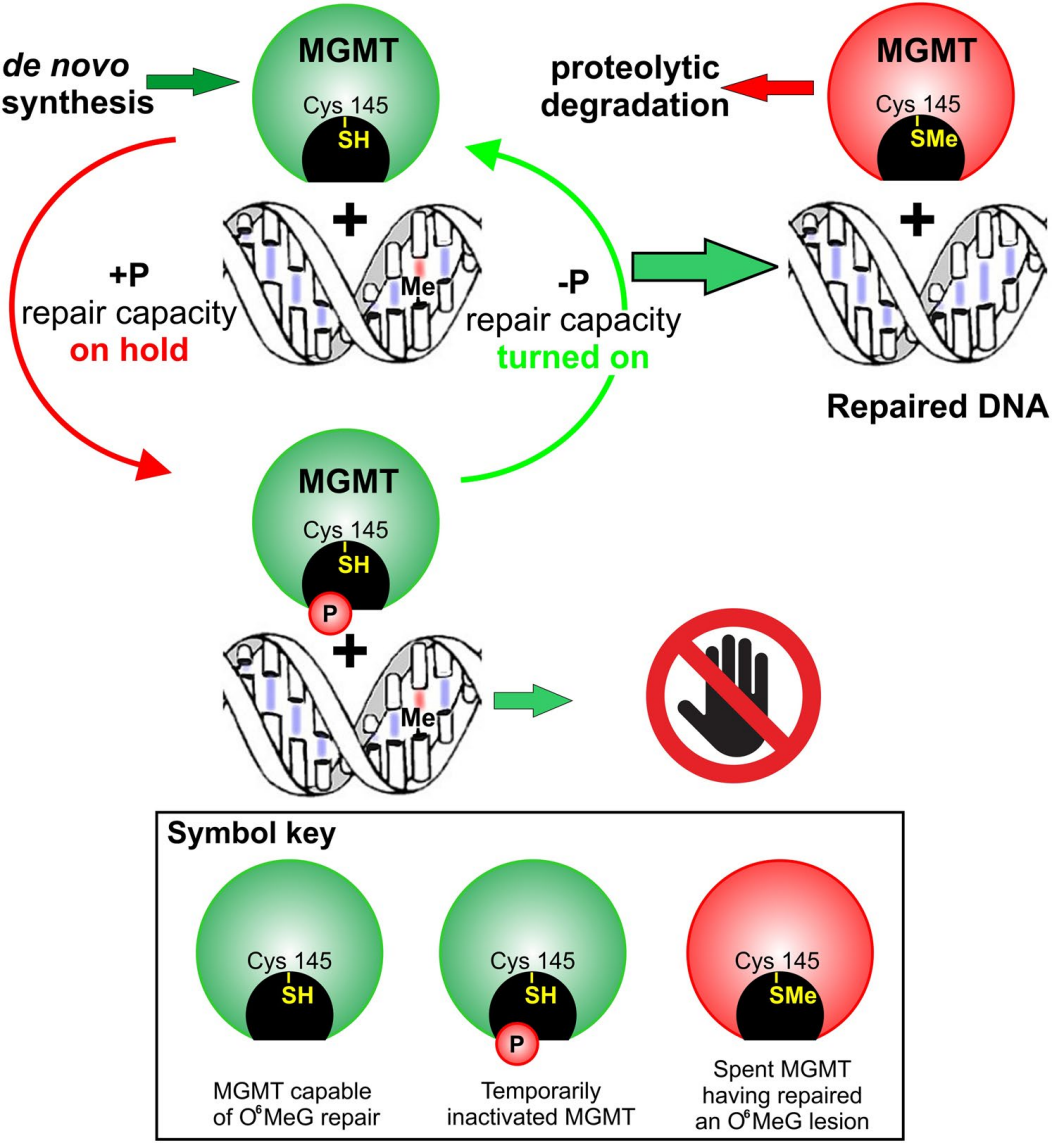
The blockade of MGMT activity by phosphorylation placing this protein ‘On Deck’ or ‘In The Hole’, and dephosphorylation placing this protein in the ‘Up’ position

One could speculate on the behaviour of mismatch repair (MMR) in normal healthy cells; that MMR would be given a set number of repair attempts before this approach was abandoned (this would circumvent the cell becoming trapped in a futile (Zhang et al. 2012) cycle (Fig. 4) and the cell switched to another repair system such as MGMT which was previously 'On deck', this switching is illustrated in (Fig. 5); thus preventing the cell becoming locked in the aforementioned futile cycle, as occurs in some cancerous cells in the absence of MGMT, resulting in the triggering of an apoptotic switch (programmed cell death) (Tang et al. 2019).

Proteins undergoing multisite phosphorylation have recently been demonstrated to play an important role in cellular signal processing by cyclin-dependant kinase 1 (Valk et al. 2014). Cyclin-dependant kinase 1 is involved in the control of cellular division and differentiation using phosphologic type gates (Valk et al. 2014), and this leads to the speculation that phosphologic gates are used in the control of many aspects of cellular activity beyond the control of DNA repair.

## Declarations

### Data availability

No materials were used to acquire the information provided in this manuscript, and no experiments were performed by the author to produce this manuscript.

### Code availability

No code has been utilized or described in this article. Thus, none is available.
